# Spastic Esophageal Disorders—How Much Muscle Should We Cut?

**DOI:** 10.1111/den.15068

**Published:** 2025-06-10

**Authors:** Siew‐Fung Hau, Hon Chi Yip

**Affiliations:** ^1^ Department of Surgery, Faculty of Medicine The Chinese University of Hong Kong Hong Kong SAR Hong Kong

Determining the best approach for treating spastic esophageal disorder (SED) remains a challenge because of its rarity. The retrospective analysis by Tatsuta et al. offered valuable insights into the effectiveness and safety of peroral endoscopic myotomy (POEM) for conditions, such as type III achalasia, distal esophageal spasm (DES), and jackhammer esophagus (JE) [1]. The study reported a promising clinical success rate of 92.6% at 1 year, highlighting POEM's efficacy in this group of patients. These results are consistent with meta‐analyses by Khan et al. and Chandan et al., which found POEM had a clinical success rate of 87% and 89.6% in cohorts of patients with SED, respectively [2, 3].

According to the Chicago Classification Version 4.0, DES and JE typically have normal integrated relaxation pressure (IRP) across the lower esophageal sphincter (LES) [4]. Therefore, the LES could theoretically be preserved during POEM in order to reduce the incidence of post‐POEM gastroesophageal reflux disease (GERD) in these patients. The current study showed that the overall rate of erosive esophagitis was 27.7% at 2–3 months and 16.1% at 1 year. The patient group (DES and JE) with LES‐preserving POEM showed a non‐significant trend toward less erosive esophagitis (16.7% at 2–3 months, 20% at 1 year) while the treatment efficacy was similar. Although the size of the cohort was small, with only seven patients, this finding provided valuable direction for future treatments. Perananthan et al. also reported on 16 cases of LES‐preserving POEM in DES and hypercontractile esophagus (HE), achieving clinical success in 93.8% of patients [5]. This approach reduced post‐POEM esophagitis to 12.5%, compared with the usual rate of around 30% reported in the literature.

Currently, there are only small case series on LES‐preserving POEM as a treatment strategy for SED, so the evidence is still limited. The latest study added to the existing literature and showed promising outcomes with LES preservation in terms of post‐procedure GERD. However, the follow‐up period was short and only 1‐year outcome was reported. Concerns about preserving the LES include the potential for an imbalance between the propulsive strength of the dissected esophagus and the normal pressure of the undissected LES, which could cause dysphagia [5]. Additionally, there were data in the literature showing a small proportion of patients with DES progressing to type III achalasia (8%). This may support the inclusion of LES division in the initial POEM [6]. Another potential issue with LES preservation would be the development of blown‐out myotomy, a condition that manifests as a focal dilatation of the distal esophagus after treatment with POEM. In a recent study focusing on patients who underwent POEM for achalasia, 31.3% of the patients developed this complication upon 5‐year follow‐up [7]. A long myotomy and abnormal residual esophagogastric junction tone were identified as possible risk factors for its development [8]. A longer term follow‐up on the subgroup of patients with POEM and LES preservation is thus desired.

Given the limited data and sample size, it is difficult to draw any definitive conclusions at the mean time. Further large‐scale studies and randomized controlled trials would be needed to guide treatment strategies.

## Author Contributions

S.‐F.H. is responsible for literature review and writing – original draft preparation. H.C.Y. is responsible for conceptualization and writing – review and editing.

## Conflicts of Interest

The authors declare no conflicts of interest.

## Linked Articles

Per‐oral endoscopic myotomy in spastic esophageal disorders: Clinical outcomes and optimal approaches. https://doi.org/10.1111/den.15008
